# Perioperative Immunotherapy for Pancreatic Cancer: A Systematic Review of Randomized Controlled Trials

**DOI:** 10.1007/s12029-026-01431-z

**Published:** 2026-03-03

**Authors:** Tanzeela Sameen Saeed, Muhammad Ramish Saeed, Muhammad Shoaib Qureshi, Nihal Habib, Uswa Ashraf, Sama Mehtab, Muhammad Fahad Abdullah, Mirza Farhana Iqbal Chowdhury, Armeen Saeed, Khizar Razzaq, Muhammad Asif Maqbool

**Affiliations:** 1 Services Institute of Medical Sciences, Lahore, Pakistan; 2 King Edward Medical University, Lahore, Pakistan; 3 Allama Iqbal Medical College, Lahore, Pakistan; 4 Department of Surgery, Johns Hopkins University School of Medicine, Baltimore, MD USA; 5 North Dakota State University, Fargo, ND USA; 6 North Surgical Ward, Department of Surgery, Mayo Hospital, King Edward Medical University, Lahore, Pakistan

**Keywords:** Pancreatic cancer, Immunotherapy, Perioperative, Surgery, Systematic Review, PDAC

## Abstract

**Purpose:**

Pancreatic cancer poses a significant clinical challenge due to its aggressive nature and poor prognosis. While surgery remains the primary curative option, the emergence of immunotherapy presents a promising avenue for improving patient outcomes. This systematic review evaluates the effectiveness and safety of perioperative immunotherapy in patients with pancreatic cancer.

**Methods:**

PubMed, Cochrane Library, and clinicaltrials.gov were searched for all relevant articles published up to July 2024. Only randomized controlled trials (RCTs) were included, and the PRISMA guidelines were followed. The primary outcome was overall survival (OS); secondary outcomes included progression-free survival (PFS), recurrence-free survival (RFS), disease-free survival (DFS), adverse events, and immune activity.

**Results:**

A total of six RCTs were included in this review. Among the six included trials, three reported significant improvements in OS with perioperative immunotherapy. PFS was improved in three studies, while RFS and DFS showed mixed results; only one trial reported a significant improvement in DFS. Safety outcomes varied across studies, with one study reporting Grade ≥ 3 adverse events related to immunotherapy, while two studies found that the side effects were comparable between the two groups. Notably, four studies observed increased immune activity, marked by higher lymphocyte counts and enhanced activity of tumor-infiltrating lymphocytes in the immunotherapy group.

**Conclusion:**

Perioperative immunotherapy appears to be a feasible and potentially beneficial approach in pancreatic cancer, showing promise in improving survival and immune responsiveness. While findings are heterogeneous, these results support further investigation through large-scale, biomarker-driven studies to optimize its integration into perioperative management strategies.

**Supplementary Information:**

The online version contains supplementary material available at 10.1007/s12029-026-01431-z.

## Introduction

In the current era of therapeutic advancement, pancreatic carcinoma (PC) remains the third leading cause of cancer-related deaths in the US, with the highest mortality rate of all the major cancers [1]. Even though the recent American Cancer Society’s Cancer Facts & Figs. 2024 Report shows an increase in the five-year survival rate following surgery, it is still at 13%, and PC is projected to become the second leading cause of cancer-related deaths before 2030 [1, 2].

Pancreatic adenocarcinoma, derived from the glandular tissue of the pancreas, forms a predominant part of PC cases. Due to inconspicuous symptoms, the majority of PC patients present with locally advanced or metastatic disease, where treatment relies on chemotherapy and radiation with limited benefit [3]. Additionally, patients undergoing surgery face unfavorable outcomes due to the high rate of recurrence and distant metastases attributed to the positive resection margin and the highly aggressive nature of the cancer [4]. This underscores the need for innovative strategies to improve outcomes in this lethal disease.

In recent years, the focus has been shifted to immunotherapy, which boosts the body’s immune system to target and eliminate tumors through exogenous immune cells, activation of endogenous immune cells, or modulation of immune pathways [5]. Immunotherapy has emerged as a promising approach for treating solid tumors like Melanoma and Non-Small Cell Lung Carcinoma (NSCLC), offering potential long-lasting responses and even curative outcomes [6]. However, its use in pancreatic cancer faces challenges due to poor antigenicity, dense desmoplastic stroma, and PC’s immune-privileged tumor microenvironment (TME) with prominent M2 macrophages and regulatory T-cells that limit cell-mediated immunity, making PC an immunologically “cold” tumor [7].

The perioperative period represents a critical window in cancer treatment, where interventions could potentially alter the course of disease progression. Perioperative immunotherapy for pancreatic cancer is an emerging area of research. While surgery remains the mainstay of curative treatment, the use of immunotherapy in neoadjuvant or adjuvant settings can improve surgical outcomes by early treatment of micrometastatic disease, reducing tumor size, and achieving a margin-negative resection, thus decreasing post-surgical recurrence [8]. Further, immunotherapy administered before surgery has the potential to reduce perioperative immunosuppression and enhance immune surveillance by better identifying tumor antigens [9].

With recent clinical trials showing improvement in survival outcomes with the use of immunotherapeutic agents in the perioperative context and earlier trials showing treatment response variability, there is a need for a systematic analysis of existing evidence. Therefore, the purpose of this systematic review of randomized controlled trials (RCTs) is to evaluate the effectiveness and safety of perioperative immunotherapy in improving survival outcomes and enhancing immune responses in patients with pancreatic cancer. Understanding the impact of perioperative immunotherapy can inform clinical decision-making and, with further research, guide the integration of immunotherapy into the standard care for pancreatic cancer patients.

## Materials and Methods

### Study Design and Protocol

Our study involved a systematic and detailed review of relevant literature sourced from various databases to assess the safety and effectiveness of perioperative immunotherapy in improving survival outcomes and enhancing immune responses in patients with pancreatic cancer. The study was conducted and reported according to the Preferred Reporting Items for Systematic Reviews and Meta-Analyses (PRISMA) 2020 protocols [10]. The review protocol was prospectively registered in the International Prospective Register of Systematic Reviews (PROSPERO) under the registration number CRD42024613110.

### Search Strategy

A comprehensive literature search was conducted on PubMed, Cochrane Library, and Clinicaltrials.gov for studies published up to July 2024. The search strategy combined Medical Subject Headings (MeSH) terms and keywords related to pancreatic cancer and immunotherapy, including various terms for pancreatic tumors and different types of immunotherapy: Pancreatic Neoplasms [Mesh], pancreatic cancer, pancreatic adenocarcinoma, pancreatic tumor, pancreatic carcinoma, Pancreas Neoplasms, Cancer of Pancreas, Pancreas Cancers, Pancreatic Acinar Carcinomas, Neoadjuvant Immunotherapy, Preoperative Immunotherapy, Perioperative Immunotherapy, Adjuvant Immunotherapy, Postoperative Immunotherapy (Supplementary Material 1). In addition, the reference lists of previous reviews were manually screened to identify additional eligible studies.

### Eligibility Criteria

The studies published in English were included based on PICOS criteria:

- Participants (P): Patients with histologically confirmed pancreatic cancer (all histologic types and stages).
- Intervention (I): Perioperative immunotherapy, including neoadjuvant, adjuvant, or perioperative use of immunotherapy agents (such as checkpoint inhibitors, monoclonal antibodies, or other immunomodulatory drugs).
- Comparator/Control (C): Standard treatment, including surgery, chemotherapy, radiotherapy, or a combination of these interventions.
- Outcomes (O): Overall survival (OS), recurrence-free survival (RFS), progression-free survival (PFS), disease-free survival (DFS), adverse effects, and immune activity.
- Study design (S): RCTs.

Consequently, non-randomized studies, case reports, case series, reviews, meta-analyses, editorials, animal studies, and studies with incomplete data or unclear reporting of outcomes were excluded.

### Study Selection

All the articles retrieved from the databases mentioned above were screened after the removal of duplicate publications. Initially, two reviewers independently screened the titles and abstracts of all studies. Those articles meeting the inclusion criteria were subjected to a full-text review for final eligibility assessment. Any differences of opinion during either stage of screening were resolved through mutual discussion or consultation with a third reviewer.

### Data Extraction

For each study included in the review, the data were systematically extracted by two independent reviewers. A standardized data extraction form was used, which included the following variables:

- Study characteristics (first author, publication year, country).
- Patient characteristics (population type, sample size).
- Intervention details (type of immunotherapy, timing: neoadjuvant/adjuvant).
- Comparator arm.
- Clinical outcomes: OS, PFS/DFS, recurrence, and adverse events.
- Immunologic and biomarker findings (e.g., PD-L1 status, ELISpot response, TIL density).
- Duration of follow-up.
- Methodological quality indicators.

Extracted data were cross-checked for consistency, and discrepancies were sorted by re-evaluation and agreement.

### Outcome Assessment

The outcomes assessed in this review included primary and secondary measures to evaluate the effectiveness and safety of perioperative immunotherapy in pancreatic cancer. The primary outcome assessed was overall survival (OS), defined as the time from treatment initiation to death. Secondary outcomes included progression-free survival (PFS), measuring the time to disease progression or death, disease-free survival (DFS), indicating the duration without detectable disease, and recurrence-free survival (RFS), the time until cancer recurrence or death. Safety was evaluated by analyzing adverse effects using reported toxicity grading scales. Immune activity was assessed through biomarkers such as immune cell infiltration and cytokine levels.

### Quality Assessment

The methodological quality of the included randomized controlled trials was independently assessed by two reviewers using the Cochrane Risk of Bias 2.0 (RoB 2) tool [11]. Any disagreements were resolved through discussion and, when necessary, consultation with a third reviewer. The RoB 2 tool evaluates five key domains: bias arising from the randomization process, deviations from intended interventions, missing outcome data, outcomes measurement, and selection of the reported results. Each domain was assigned a risk level of “low risk,” “some concerns,” or “high risk.” An overall risk of bias judgment was then determined for each study: low risk if all domains were judged to be low, high risk if one or more domains had high risk, or if multiple domains raised concerns that substantially lowered confidence in the results. The findings of the risk of bias assessment are presented descriptively.

## Results

### Study Selection

A total of 2,866 records were identified through database and manual searches, including PubMed (*n* = 2,638), Cochrane Library (*n* = 157), ClinicalTrials.gov (*n* = 15), and references of previous reviews (*n* = 56). After removing 100 duplicate records and 5 retracted articles, 2,761 records were screened based on titles and abstracts. Of these, 2,707 records were excluded during the primary screening phase.

Fifty-four full-text reports were sought for retrieval, of which 8 could not be retrieved. The remaining 46 articles were assessed for eligibility. Among these, 40 studies were excluded for the following reasons: immunotherapy used in both study arms (*n* = 8), lack of immunotherapy intervention (*n* = 2), not randomized controlled trials (*n* = 27), or absence of the desired outcomes (*n* = 3).

**Fig. 1:**
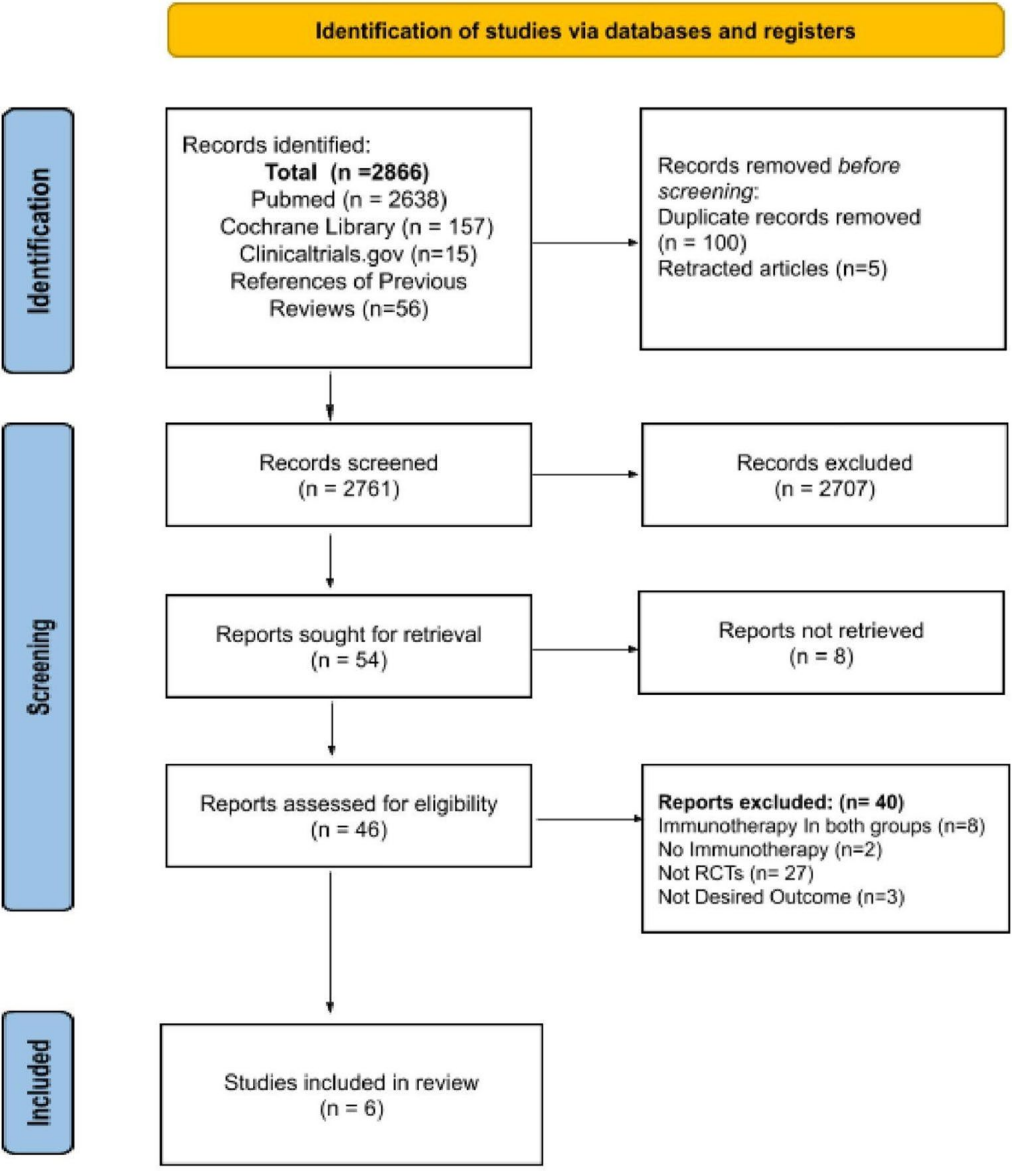
PRISMA flow diagram for the study selection

Ultimately, 6 randomized controlled trials (RCTs) [12–17] met the inclusion criteria and were included in this systematic review. A PRISMA flow diagram illustrating the selection process is presented in Fig. 1.

### Study Characteristics

A total of six randomized controlled trials (RCTs), published between 2000 and 2023, were included in this review. The studies were conducted across a range of countries, including the United States, Italy, Japan, China, Greece, and a multi-country cohort, and enrolled a total of 548 patients with resectable, borderline resectable, or locally recurrent pancreatic cancer. All studies evaluated perioperative immunotherapy interventions in either the neoadjuvant or adjuvant setting.

The types of immunotherapy varied across studies and included:

- Interleukin-2 (IL-2) cytokine therapy,
- Lymphokine-activated killer (LAK) cell therapy,
- Cancer vaccines (GI-4000 expressing RAS mutations),
- Immune checkpoint inhibitors (anti-PD-1 pembrolizumab),
- Combination immunotherapy with targeted therapy (MEK inhibitor trametinib).

The interventions were combined with surgery, chemotherapy, and/or radiotherapy, and compared to standard treatment arms, including surgery alone, chemoradiotherapy, or placebo.

Follow-up periods ranged from 13 months to 60 months. Outcomes assessed across studies included overall survival (OS), progression-free survival (PFS) or disease-free survival (DFS), recurrence patterns (especially hepatic metastasis), and treatment-related adverse events. Some studies also assessed immune-related biomarkers, such as tumor-infiltrating lymphocytes (TILs), regulatory T-cell (Treg) suppression, and response to RAS peptides or PD-L1 expression. A detailed summary of the study characteristics is presented in Table 1.

**Table 1:** Characteristics of the RCTs included in the review

Study	Design	Country	Sample Size	Population	Intervention	Comparator	Timing	Immunotherapy Type	Follow-up Duration
Caprotti et al. (2008) [12]	RCT	Italy	30	Resectable PDAC	IL-2 + Melatonin (3 days pre-op)	Surgery alone	Neoadjuvant	IL-2 (cytokine)	36 months
Katz et al. (2023) [13]	RCT (Phase Ib/II)	USA	37	Resectable/Borderline PDAC	Neoadj CRT + Pembrolizumab	Neoadj CRT	Neoadjuvant	PD-1 inhibitor	Not Reported
Kobari et al. (2000) [14]	RCT	Japan	30	Resectable PDAC	Adjuvant LAK cell infusion via the portal vein	Surgery alone	Adjuvant	Adoptive cell therapy	30.8 months
Lygidakis et al. (2002) [15]	RCT	Greece	128	Stage III PDAC	Surgery + regional chemo + IL-2	Surgery / Surgery + chemo	Adjuvant	IL-2 (locoregional)	5 years
Muscarella et al. (2021) [16]	RCT (Phase 2, DB)	USA + Int’l	176	Resected PDAC, RAS-mutated	GI-4000 (yeast vaccine) + Gemcitabine	Placebo + Gemcitabine	Adjuvant	RAS peptide vaccine	60 months
Zhu et al. (2023) [17]	RCT (Phase 2)	China	147	Locally recurrent PDAC, KRAS-mut, PD-L1+	SBRT + Pembrolizumab + Trametinib	SBRT + Gemcitabine	Adjuvant/recurrence	PD-1 + MEK inhibitor	13.1 months

### Risk of Bias Assessment

**Fig. 2:**
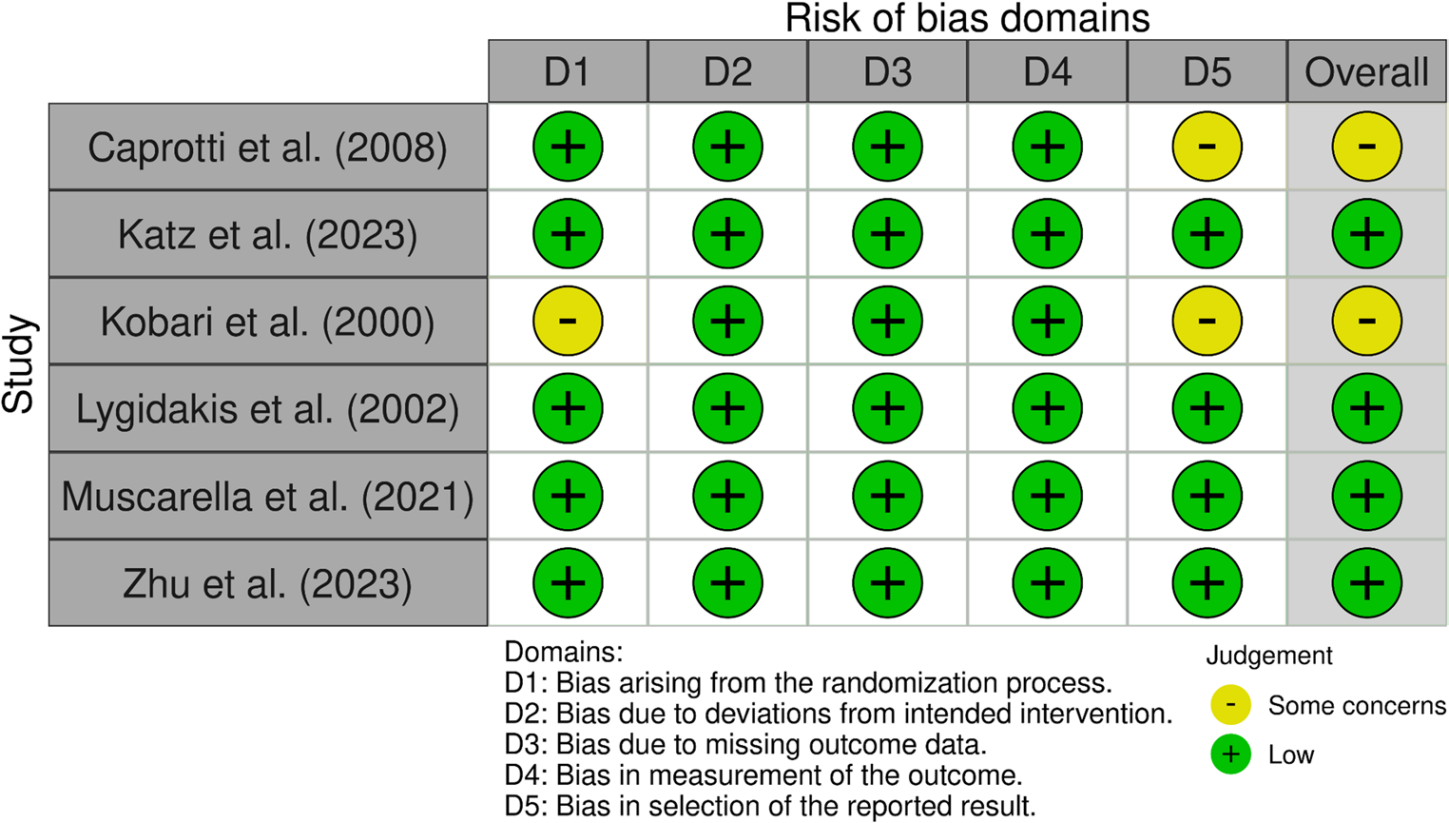
Risk of Bias Assessment in the included RCTs

The risk of bias was evaluated using the Cochrane Risk of Bias 2.0 tool across five domains. Four studies were assessed as having low risk of bias in all domains [13, 15–17]. Two studies (Caprotti et al., Kobari et al.) were judged to have “some concerns” primarily due to unclear details in the randomization process or selective reporting [12, 14]. A summary of the risk of bias assessment is presented in Fig. 2.

### Outcome Synthesis

#### Progression-Free Survival (PFS), Recurrence-Free Survival (RFS), and Disease-Free Survival (DFS)

Progression-Free Survival (PFS) outcomes were reported in three of the six included trials. Zhu et al. (2023) demonstrated a statistically significant improvement in PFS with the combination of stereotactic body radiotherapy (SBRT), pembrolizumab, and trametinib compared to SBRT plus gemcitabine (median PFS: 8.6 vs. 5.0 months; *p* = 0.0021) [17]. Similarly, Katz et al. (2023) reported a numerical improvement in PFS with the addition of pembrolizumab to neoadjuvant chemoradiotherapy (18.2 vs. 14.1 months), although this difference did not reach statistical significance [13]. Caprotti et al. (2008) reported a significant increase in the “free-from-progression period” (FFPP), a proxy for PFS, in patients receiving short-course preoperative IL-2 (*p* < 0.01), suggesting a delay in disease progression [12].

Recurrence-Free Survival (RFS) was reported in two of the six trials. Katz et al. (2023) documented a median RFS of 22.3 months in the pembrolizumab arm (Arm A) and 21.8 months in the control arm (Arm B), indicating no significant difference in recurrence-free outcomes between groups. Despite a higher R0 resection rate in the immunotherapy group, this did not translate into improved RFS [13]. Muscarella et al. (2021) also reported RFS, finding no significant difference between the GI-4000 vaccine and placebo arms (median RFS: 354 vs. 357 days), despite a higher immunologic response in certain subgroups [16].

Disease-Free Survival (DFS) was assessed in only one trial. Lygidakis et al. (2002) demonstrated significantly improved DFS in the chemoimmunotherapy group (26.7 months) compared to chemotherapy alone (21.5 months) and surgery alone (14.2 months), indicating improved disease control with immunomodulation [15].

Collectively, these findings highlight the potential of perioperative immunotherapy to improve PFS, DFS, and RFS in pancreatic cancer, particularly when integrated with radiotherapy or targeted agents. However, the heterogeneity in outcome definitions and reporting across studies limits comparability and underscores the need for standardized endpoints in future trials.

#### Recurrence Patterns

Three studies specifically analyzed recurrence patterns. Kobari et al. (2000) showed that the liver was the most common site of recurrence, but hepatic metastases were significantly less frequent in the LAK group (13%) compared to the control (47%) [14]. Lygidakis et al. (2002) found a substantial reduction in locoregional (15%), liver (12%), and peritoneal (19%) recurrence in the IL-2 chemoimmunotherapy arm versus the control groups [15]. Katz et al. (2023) reported a higher R0 resection rate in the immunotherapy group (82% vs. 57%), possibly contributing to delayed recurrence, although specific recurrence sites were not described [13].

#### Immune Biomarkers and Immunologic Findings

Four studies included exploratory immune or biomarker analyses. Caprotti et al. (2008) found that IL-2 therapy prevented postoperative lymphocytopenia and significantly increased circulating lymphocyte counts. However, histological examination did not reveal increased intratumoral lymphocyte infiltration [12]. Katz et al. (2023) reported numerically higher CD8⁺ and CD4⁺ T-cell densities and reduced immunosuppressive myeloid cells in the pembrolizumab group, but differences were not statistically significant. PD-L1 expression was uniformly low across tumors in both arms, consistent with the known immune resistance of pancreatic cancer [13]. Lygidakis et al. (2002) reported increased leukocyte infiltration in tumors and fewer postoperative infections following regional IL-2 therapy, suggesting local immune activation despite the absence of quantitative immune profiling [15]. Muscarella et al. (2021) reported stronger vaccine-specific T-cell responses and significant suppression of regulatory T cells in R1-resected patients treated with GI-4000 [16].

#### Safety and Adverse Events

Immunotherapy was generally well tolerated, and safety outcomes varied across all trials. Katz et al. (2023) and Zhu et al. (2023) reported slightly higher rates of Grade ≥ 3 adverse events in the immunotherapy arms (38% vs. 31% and 28.6% vs. 19.0%, respectively), with most events being hematologic and gastrointestinal symptoms. However, these did not lead to treatment discontinuation and were considered manageable [13, 17].

Muscarella et al. (2021) found the GI-4000 vaccine to be well-tolerated, with only mild injection site reactions, hematologic toxicities (anemia, neutropenia), and no unexpected safety signals [16]. Similarly, Caprotti et al. (2008) and Lygidakis et al. (2002) reported no severe toxicities, with IL-2-associated side effects limited to low-grade fever, mild hematologic abnormalities, and catheter complications [12, 15]. Kobari et al. (2000) did not report any adverse event data [14]. All outcomes are summarized in Table 2.

**Table 2:** Outcomes Summary Table

Study	OS Outcome	PFS	RFS	DFS	Biomarker/Immune Data	Toxicity/Safety
Caprotti et al. (2008) [12]	↑ OS (*p* < 0.05)	↑ FFPP (*p* < 0.01)	Not reported	Not reported	↑ post-op lymphocytes; no TILs	Fever, fatigue; No IL-2-related toxicity
Katz et al. (2023) [13]	27.8 vs. 24.3 mo (NS)	18.2 vs. 14.1 mo (NS)	22.3 vs. 21.8 mo (NS)	Not reported	↑ CD8 + and CD4⁺ T-cells (NS); low PD-L1	Grade ≥ 3 AEs: 38% vs. 31%
Kobari et al. (2000) [14]	↑ OS (*p* = 0.02)	Not reported	Not reported	Not reported	Not reported	No AE data provided
Lygidakis et al. (2002) [15]	31.1 vs. 25.0 vs. 18.8 mo	Not reported	Not reported	26.7 vs. 21.5 vs. 14.2 mo	↑ Leukocyte infiltration	Fever, catheter issues, mild hematologic toxicity
Muscarella et al. (2021) [16]	698 vs. 751 days (NS)	Not reported	354 vs. 357 days (NS)	Not reported	↑ T-cell response and ↓ Treg in R1 pts	Injection site reaction, anemia, neutropenia
Zhu et al. (2023) [17]	15.1 vs. 12.4 mo (*p* = 0.071)	8.6 vs. 5.0 mo (*p* = 0.0021)	Not reported	Not reported	Not reported	Grade 3–4 AEs: 28.6% vs. 19.0%

#### Overall Survival (OS)

All six included randomized controlled trials reported overall survival (OS) as a primary or secondary outcome, although statistical significance varied. Three out of six RCTs demonstrated improvements in Overall Survival (OS) for the immunotherapy group, while the other three studies did not show significant improvements.

Caprotti et al. (2008) demonstrated a significant improvement in OS with a short preoperative course of subcutaneous interleukin-2 (IL-2) combined with melatonin in patients undergoing resection for pancreatic ductal adenocarcinoma. Patients receiving IL-2 had a statistically longer OS compared to those undergoing surgery alone (*p* < 0.05) [12]. Similarly, Kobari et al. (2000) reported a significant OS benefit (*p* = 0.02) with the use of intraportal adoptive immunotherapy using lymphokine-activated killer (LAK) cells following surgery [14]. Lygidakis et al. (2002) also reported a favorable OS trend, with the chemoimmunotherapy group (surgery + regional chemotherapy + IL-2) achieving a mean OS of 31.1 months compared to 25.0 months in the chemotherapy-alone group and 18.8 months in the surgery-alone group [15].

In contrast, more recent studies involving immune checkpoint inhibitors and cancer vaccines did not show statistically significant OS benefits. Muscarella et al. (2021) found no difference in median OS between patients treated with the GI-4000 RAS peptide vaccine plus gemcitabine (698 days) and those receiving placebo plus gemcitabine (751 days) [16]. Similarly, Katz et al. (2023) reported comparable OS between neoadjuvant chemoradiotherapy (CRT) plus pembrolizumab (27.8 months) and CRT alone (24.3 months) [13]. Zhu et al. (2023) observed a modest numerical improvement in OS with the combination of stereotactic body radiotherapy (SBRT), pembrolizumab, and trametinib (15.1 vs. 12.4 months), though the difference did not reach statistical significance (*p* = 0.071) [17].

Taken together, early studies involving IL-2 and LAK therapy demonstrated statistically significant OS benefits, while more recent trials using checkpoint inhibitors and peptide vaccines showed limited survival gains. Overall, three studies showed favorable OS trends with perioperative immunotherapy, though only two demonstrated statistical significance.

## Discussion

This systematic review identified six randomized controlled trials (RCTs) evaluating perioperative immunotherapy in patients with resectable or borderline resectable pancreatic ductal adenocarcinoma (PDAC). Collectively, these studies demonstrate that immune-based interventions can be safely integrated into the perioperative treatment paradigm and may induce measurable immunologic activity. However, evidence for consistent and clinically meaningful survival benefit remains limited, highlighting the exploratory nature of the current data and the need for further rigorous investigation.

A key consideration in interpreting these findings is the substantial heterogeneity of immunotherapeutic strategies across included trials. Interventions ranged from early cytokine-based therapies and adoptive cell therapy to cancer vaccines and immune checkpoint inhibitors combined with chemotherapy or radiotherapy. These modalities differ fundamentally in mechanism of action, toxicity profile, logistical feasibility, and biological rationale. Consequently, direct comparisons across studies are inappropriate, and observed differences in outcomes likely reflect both mechanistic diversity and temporal evolution of standard-of-care treatments rather than superiority of any single approach.

In our review, three of the six studies employed cytokine-based immunotherapy using interleukin-2 (IL-2) or lymphokine-activated killer (LAK) cells. These early randomized trials using interleukin-2 (IL-2)-based or adoptive immunotherapy demonstrated statistically significant improvements in overall survival (OS) or disease-free survival (DFS). Caprotti et al. demonstrated a statistically significant improvement in overall survival (OS) and free-from-progression period (FFPP) with the use of preoperative IL-2 and melatonin [12]. Similarly, Kobari et al. found that intraportal delivery of LAK cells significantly reduced hepatic metastasis and improved OS [14]. Lygidakis et al. used regional IL-2 in combination with chemotherapy post-resection, reporting significant reductions in locoregional and distant recurrence along with prolonged disease-free survival (DFS) [15]. These findings are biologically supported by preclinical work showing that IL-2 and LAK cells can enhance cytotoxic T-cell activation and tumor clearance in solid tumors [18, 19]. These studies suggest that perioperative immune stimulation may influence early metastatic spread and recurrence patterns in PDAC.

Nevertheless, none of these early cytokine-based or adoptive immunotherapy regimens have been evaluated in contemporary randomized trials, raising concerns regarding scalability, reproducibility, and comparative effectiveness. Their absence from modern clinical development likely reflects logistical complexity, limited therapeutic indices, and the emergence of more targeted immunotherapeutic strategies.

In contrast, more recent randomized trials have explored modern immunotherapies such as immune checkpoint inhibitors and vaccine-based approaches. Muscarella et al. evaluated the GI-4000 yeast-based RAS vaccine in the adjuvant setting. Although no survival benefit was observed in the overall cohort, immune activation, including ELISpot positivity and regulatory T-cell suppression, was more prominent in patients with R1 resections and those with the BDX-001 proteomic classifier [16]. Similarly, Katz et al. added pembrolizumab to neoadjuvant chemoradiotherapy (CRT), achieving improved R0 resection rates and PFS, but without a statistically significant OS gain [13]. Zhu et al. investigated a novel combination of pembrolizumab and the MEK inhibitor trametinib following stereotactic body radiotherapy (SBRT). They found that dose-escalated SBRT improved PFS significantly compared to gemcitabine (8.6 vs. 5.0 months, *p* = 0.0021), although OS differences were not statistically significant [17].

These findings align with broader literature indicating that pancreatic ductal adenocarcinoma (PDAC) is unlikely to respond to checkpoint inhibition alone [20, 21]. This resistance has been attributed to a dense desmoplastic stroma, an immunosuppressive tumor microenvironment, and a low mutational burden, and emerging strategies aim to overcome these barriers by combining immunotherapy with standard perioperative treatments [22–24]. Radiotherapy and MEK inhibition may sensitize PDAC to immunotherapy by increasing antigen presentation and reversing immune exclusion [25]. The integration of such multimodal approaches may enhance immune infiltration, as suggested by Zhu et al. [17] and Bear et al. [7]. However, although combination strategies incorporating chemotherapy, radiotherapy, or targeted agents may modulate stromal barriers and sensitize tumors to immune-mediated killing, durable survival benefits have yet to be consistently demonstrated in randomized perioperative settings.

Across all included trials, perioperative immunotherapy was generally well tolerated and did not compromise surgical feasibility or delay standard oncologic treatments. Early studies using IL-2 or LAK therapy reported minimal toxicity, mostly low-grade flu-like symptoms. In contrast, modern checkpoint and vaccine trials reported manageable but slightly higher rates of grade ≥ 3 adverse events [13, 16, 17]. Importantly, none of the trials reported prohibitive perioperative morbidity attributable to immunotherapy, supporting the safety of immune modulation during the perioperative window.

Immune correlative analyses, although inconsistently reported, provided valuable biological insights. Included studies demonstrated treatment-related immune activation, including enhanced tumor-infiltrating lymphocytes, vaccine-specific T-cell responses, and modulation of regulatory immune populations. Biomarker analyses were limited but suggestive of emerging precision immunotherapy strategies. Notably, the Muscarella trial highlighted the potential of immune classifiers (BDX-001) to identify responders to vaccination. Zhu et al. restricted inclusion to patients with KRAS mutations and PD-L1 positivity, which may partly explain the observed immunologic and clinical benefits. Collectively, these findings reflect a shift toward biomarker-guided perioperative immunotherapy in PDAC. However, the lack of standardized immune endpoints, heterogeneous biomarker selection, and inconsistent reporting across studies limit cross-trial comparisons and preclude definitive conclusions regarding predictive immune biomarkers, underscoring the need for harmonized correlative frameworks in future randomized trials [26].

Ongoing perioperative immunotherapy trials increasingly reflect lessons learned from the studies summarized in this review, incorporating modern chemotherapy backbones, radiation sensitization, biomarker-driven enrollment, and rational combinatorial strategies. Although ongoing trials were screened through ClinicalTrials.gov, they were excluded from this review due to the absence of reported outcome data. Their design nevertheless underscores the evolving direction of the field toward mechanism-based and personalized immunotherapy strategies in PDAC.

Recent advances in personalized neoadjuvant treatment strategies, individualized vaccine platforms, and therapeutic modulation of the tumor microenvironment represent important and rapidly evolving directions in pancreatic cancer management. Biomarker-informed selection of neoadjuvant systemic therapy has demonstrated feasibility and may improve treatment completion and resectability in localized disease [27]. In parallel, personalized neoantigen-based vaccine approaches have shown the capacity to induce robust tumor-specific T-cell responses and suggest a potential association between vaccine-induced immunity and delayed recurrence [28]. Additionally, emerging strategies aimed at remodeling the immunosuppressive tumor microenvironment, particularly through targeted disruption of myeloid-derived suppressor cell-mediated immune tolerance, have demonstrated promising preclinical and early translational activity [29]. However, despite their strong biological rationale and encouraging early signals, these approaches currently lack completed randomized controlled trial data and therefore fall outside the scope of this review. Their exclusion reflects limitations in the existing evidence base rather than a lack of scientific relevance.

Several limitations of this systematic review should be acknowledged. The included randomized controlled trials were small, heterogeneous, and frequently underpowered for survival endpoints, limiting the strength of efficacy conclusions. Considerable clinical heterogeneity existed across studies with respect to immunotherapeutic modality (cytokine-based therapy, vaccines, or immune checkpoint inhibitors), timing of administration (neoadjuvant versus adjuvant), and comparator regimens (surgery alone, chemotherapy, or chemoradiotherapy). Moreover, most included trials predate contemporary standard-of-care systemic therapies, such as FOLFIRINOX or gemcitabine plus nab-paclitaxel, which limits the external validity of the findings in modern clinical practice [30, 31]. Only a few studies incorporated immunologic endpoints or biomarker-guided enrollment strategies, further constraining mechanistic interpretation. Finally, randomized trials lacking a standard-treatment comparator arm, unpublished studies, and terminated trials without reported outcomes were excluded by design to preserve methodological rigor and ensure extractable clinical data. This approach may have resulted in the omission of potentially informative early-phase experiences.

In conclusion, perioperative immunotherapy in pancreatic cancer appears feasible and biologically active, with acceptable safety profiles across diverse immune-based strategies. However, definitive survival benefits remain inconsistent, and current evidence should be considered hypothesis-generating rather than practice-changing. These findings support continued evaluation of perioperative immunotherapy within well-powered, biomarker-driven randomized trials incorporating contemporary treatment backbones and standardized immune endpoints.

## Conclusion

Perioperative immunotherapy for pancreatic ductal adenocarcinoma appears to be feasible and generally well tolerated when integrated with surgical management. Randomized evidence to date demonstrates biological activity and acceptable perioperative safety across diverse immune-based strategies; however, consistent and clinically meaningful survival benefits have not been established. The substantial heterogeneity of immunotherapeutic modalities, small sample sizes, and reliance on historical treatment backbones limit definitive efficacy conclusions. Collectively, the available evidence suggests that perioperative immunotherapy remains investigational and should not yet be considered practice-changing. Emerging data indicate that rational combination strategies and biomarker-guided patient selection may be required to overcome the immunosuppressive tumor microenvironment characteristic of pancreatic cancer. Future progress in this field will depend on well-powered, contemporary randomized trials that integrate immunotherapy with modern systemic regimens, incorporate standardized immune and clinical endpoints, and prioritize precision-based approaches to patient selection. Until such data are available, perioperative immunotherapy should remain an area of active investigation rather than routine clinical practice.

## Supplementary Information

Below is the link to the electronic supplementary material.

Supplementary Material 1

## Data Availability

No datasets were generated or analysed during the current study.
