# Supplementary material for: Perioperative Immunotherapy for Pancreatic Cancer: A Systematic Review of Randomized Controlled Trials
Source: J Gastrointest Cancer. 2026 Mar 3;57(1):56. doi: 10.1007/s12029-026-01431-z (PMC12953247; doi:10.1007/s12029-026-01431-z)
Supplement: Supplementary file 1 — Supplementary Material 1 [file 12029_2026_1431_MOESM1_ESM.pdf]

Online Resource 1

Article Title: Perioperative Immunotherapy for Pancreatic Cancer: A Systematic Review of Randomized Controlled Trials

Journal Name: Journal of Gastrointestinal Cancer

Authors:

Tanzeela Sameen Saeed, Muhammad Ramish Saeed, Muhammad Shoaib Qureshi, Nihal Habib, Uswa Ashraf, Sama Mehtab, Muhammad Fahad Abdullah, Mirza Farhana Iqbal Chowdhury, Armeen Saeed, Khizar Razzaq, Muhammad Asif Maqbool

Corresponding Author:

Khizar Razzaq
North Dakota State University, Fargo, North Dakota, USA.
Email: khizar.razzaq@ndsu.edu

Full search strategy used for PubMed, Cochrane Library, and ClinicalTrials.gov.

1. PubMed

Search conducted: 17 July 2024

Results retrieved: 2,638

Search String:

((((((((((((((((("Pancreatic Neoplasms"[Mesh])) OR (pancreatic cancer)) OR (pancreatic adenocarcinoma)) OR (pancreatic neoplasm)) OR (pancreatic tumor)) OR (pancreatic carcinoma)) OR (Pancreas Neoplasms)) OR (Pancreas Neoplasm)) OR (Cancer of Pancreas)) OR (Pancreas Cancers)) OR (Cancer of the Pancreas)) OR (Pancreas Cancer)) OR (Pancreatic Cancers)) OR (Pancreatic Carcinomas)) OR (Pancreatic Acinar Carcinoma)) OR (Pancreatic Acinar Carcinomas)) AND (((((("Immunotherapy"[Mesh]) OR (Neoadjuvant Immunotherapy)) OR (Preoperative Immunotherapy)) OR (Perioperative Immunotherapy)) OR (Adjuvant Immunotherapy)) OR (Postoperative Immunotherapy))

2. ClinicalTrials.gov

Search Conducted: 17 July 2024

Results retrieved: 15

Keywords Used: Pancreatic Cancer, Pancreatic Neoplasms, Immunotherapy

Filters Applied: Completed Trials, Studies with results

3. Cochrane Library

Search Conducted: 18 July 2024

Results retrieved: 157

Search: ("Pancreatic Neoplasms"[Mesh] OR pancreatic cancer OR pancreatic adenocarcinoma OR pancreatic neoplasm OR pancreatic tumor OR pancreatic carcinoma OR Pancreas Neoplasms OR Pancreas Neoplasm OR Cancer of Pancreas OR Pancreas Cancers OR Cancer of the Pancreas OR Pancreas Cancer OR Pancreatic Cancers OR Pancreatic Carcinomas OR Pancreatic Acinar Carcinoma OR Pancreatic Acinar Carcinomas) AND ("Immunotherapy"[Mesh] OR Neoadjuvant Immunotherapy OR Preoperative Immunotherapy OR Perioperative Immunotherapy OR Adjuvant Immunotherapy OR Postoperative Immunotherapy)
